# An Update on Pemphigus Vulgaris in Pregnancy and Neonates: Management Options and Our Clinical-Laboratory Experience

**DOI:** 10.3390/medicina62010031

**Published:** 2025-12-23

**Authors:** Maksymilian Markwitz, Natalia Welc, Monika Bowszyc-Dmochowska, Magdalena Jałowska, Marian Dmochowski

**Affiliations:** 1 Autoimmune Blistering Dermatoses Section, Department of Dermatology, Poznan University of Medical Sciences, 60-355 Poznan, Poland; 2 Doctoral School, Poznan University of Medical Sciences, 60-812 Poznan, Poland; 3 Cutaneous Histopathology and Immunopathology Section, Department of Dermatology, Poznan University of Medical Sciences, 60-355 Poznan, Poland

**Keywords:** pemphigus vulgaris, pregnancy and neonates

## Abstract

*Background and Objectives*: Pemphigus vulgaris (PV) is a rare autoimmune blistering disease caused by IgG au-toantibodies against desmoglein 1 and/or desmoglein 3, leading to flaccid blisters on the skin and mucous membranes. The course of PV during pregnancy represents a special clinical challenge due to immunological changes accompanying physiological immunosuppression and the need to protect the developing fetus. *Materials and Methods*: To analyze the current state of knowledge, a literature review was performed covering the years 2015–2025. Publications describing PV diagnosed during pregnancy or in neonates were screened, and nine case reports discussing ten patients meeting the inclusion criteria were selected for detailed analysis. In this study, we also present our own clinical case of PV in pregnancy to complement the literature review and provide practical insight into disease management. *Results*: In most cases, the disease was diagnosed in the first trimester of pregnancy, and the most common symptoms were flaccid blisters and erosions of the oral mucosa. The diagnosis was confirmed by direct immunofluorescence (DIF) and ELISA testing. The first-line treatment remained systemic glucocorticosteroids (GCS), mainly prednisolone, which is considered the safest. In resistant cases, intravenous immunoglobulins (IVIg) were used, which were considered effective and safe, though their use may limit the transplacental transfer of autoantibodies to the fetus. In newborns, the symptoms rarely occurred, were mild, and resolved spontaneously. Drugs with proven teratogenic effects, such as methotrexate, cyclophosphamide, and mycophenolate mofetil, are contraindicated during pregnancy. In the case of rituximab therapy, it is recommended to postpone pregnancy for at least 12 months after the completion of treatment to minimize the potential risk of immunosuppression in the newborn. *Conclusions*: The treatment of PV during pregnancy requires close interdisciplinary cooperation. Therapy should be carefully individualized, taking into account both therapeutic efficacy and fetal safety. Perhaps then, pregnancy-related pemphigus diseases, given their peculiarities, should be classified as a distinct variety within the desmosomal type of autoimmune blistering diseases.

## 1. Introduction

Pemphigus diseases are rare autoimmune conditions belonging to the group of autoimmune blistering diseases (AIBD). AIBD predominantly targets structural proteins and constitutes a heterogeneous group of disorders characterized by the formation of blisters and their derivative lesions resulting from autoimmune damage to adhesion molecules. The IgG-mediated pemphigus diseases, including two categories, namely pemphigus foliaceus and PV, are placed within the desmosomal type of AIBD. The pemphigus foliaceus category comprises endemic, seborrheic, herpetiform, paraneoplastic, and drug-induced variants, whereas the PV category includes mucosal-dominant, mucocutaneous, and cutaneous forms, as well as vegetative, herpetiform, paraneoplastic, and drug-induced subtypes. The incidence is estimated at 0.75 to 5 cases per million people per year [1]. Mucocutaneous PV (mc PV) is characterized by the evolutionarily initial formation of fragile blisters on the skin and mucous membranes, particularly in the oral cavity. In diseases of PV category, targets located on the surface of keratinocytes [2], predominantly in the extracellular compartment of desmosomes, are affected by the autoimmune response. Diseases of the PV category are defined mainly by the presence of IgG autoantibodies against desmoglein 3, with or without concomitant IgG autoantibodies to desmoglein 1.

Cutaneous lesions in PV have a propensity for unifying seemingly diverse clinical presentations to localize around natural body orifices (nbo), including external genitals [3]. In addition, lesions in mc PV, apart from sites adjacent to nbo, affect those mucous membranes that express desmoglein 3. These remarkable nbo sites are the scalp (the parietal area), the area around the medial canthus, the concha of the auricle and the external auditory canal, the anterior nostrils, lips, the nipples of both female and male breast, the umbilicus, sacral/pilonidal/spinal dimples, anus, external female and male genitalia, and the periungual areas of fingernails and toenails, as well as the palmar surface of hands and the plantar surface of feet (13 main nbo patterns). Remarkable mucous membranes sites are conjunctiva, nasal mucosa (nasal septum), oral cavity gingivae, oral cavity hard palate, oral cavity buccal mucosa, pharynx, larynx, esophagus, cervicovaginal mucous membrane (nine main mucosal patters). The mathematical concept of the power set but excluding the empty set (no lesions) is appropriate to calculate the number of possible patterns of lesions localization. Thus, apart from the full-blown disease, there is a huge number, 4,194,303 (2^22^ − 1), of diverse patterns of lesion localizations, since grouping these 22 main patterns is possible. Remembering that diversity should facilitate diagnosing mc PV at the clinical level is crucial for the subsequent ordering of necessary immunopathological diagnostics.

Pemphigus foliaceus and PV differ in the level of skin layer separation. In PV, the separation occurs suprabasally close to the dermal–epidermal junction. PV may develop at any age but most frequently is diagnosed in the fourth to sixth decade of life. The highest risk is observed among individuals of Jewish origin, especially Ashkenazi Jews, and in populations living in the Mediterranean region [4]. This association is related to the presence of specific HLA alleles that are predisposed to the disease, such as HLA-DRB10402 in Ashkenazi Jewish populations and HLA-DRB11401/04 and HLA-DQB1*0503 in non-Jewish populations [5,6].

Importantly, pregnancy represents a unique immunological condition, in which a delicate balance between the maternal immune system and the developing immunologically foreign fetus must be maintained. This state requires the establishment of proper immune tolerance [7]. Pregnancy modulation of the maternal immune system is mainly adjustable by sex hormones and cortisol. Progesterone is primarily responsible for the suppression of Th1 and Th17 lymphocytes, and after embryo implantation, the maternal immune system shifts toward Th2 and Treg dominance, which ensures immune tolerance and normal pregnancy development [8,9]. On the other hand, such immunological tolerance may also facilitate the development of autoimmune diseases, such as PV [10]. The incidence of PV during pregnancy is difficult to estimate because many cases are not reported or reported only as individual case reports [11].

The onset of PV in neonates seems to be rarer than in their pregnant mothers. The symptoms usually only concern the skin, rare mucous membranes, and present mild intensity. Spontaneous healing follows usually in 2–3 weeks. The disease arises because of the transmission of the maternally produced antibodies by placenta [12,13]. The slight intensity of newborn symptoms may be caused by low transfer of IgG4 autoantibodies through the placenta also, the newborn’s distribution of desmoglein 1 and 3 are different in neonatal skin in comparison to adult skin [14].

Treatment of PV is primarily based on immunosuppressive therapy, the main goal of which is to inhibit the production of autoantibodies directed against desmogleins, which are responsible for the loss of intercellular adhesion within the epidermis. Unfortunately, in pregnant women, therapeutic options are considerably limited due to the necessity of protecting the developing fetus. Many drugs routinely used in non-pregnant patients exhibit teratogenic or toxic effects on the fetus, which necessitates an individualized therapeutic approach and the selection of the safest possible treatment methods, such as prednisolone or prednisone, intravenous IVIg or dapsone.

The aim of this work was to present an update covering the last decade on PV in pregnancy, neonates and to complement these findings with our own clinical case.

## 2. Methodology

**Figure 1:**
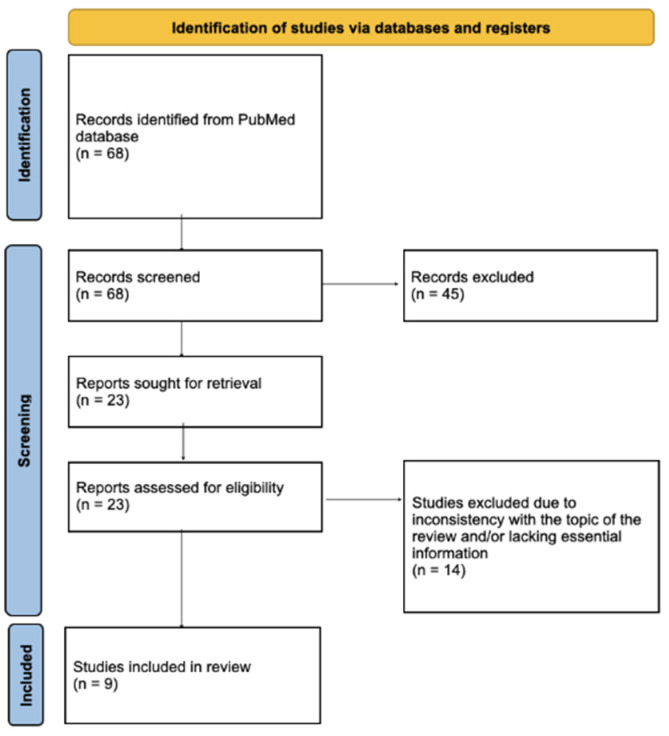
The screening and paper selection process, according to the PRISMA guideline.

Due to the limited number of published reports describing clinical features of PV during pregnancy, we decided to perform a literature review. Two authors independently screened the PubMed database by keywords “pemphigus” or “PV” and “pregnancy” for the years 2015–2025. After excluding duplicates, they identified 68 papers, 9 of which were case reports and one was a case series of two patients, altogether discussing 10 patients who were relevant to the subject of our review and included all necessary information, such as the diagnosis of PV before conception, age, gravida and para, and gestational week of the first symptoms, as well as symptoms in the mother and the neonate. Paraneoplastic pemphigus, pemphigus foliaceus, congenital bullous syphilis, pemphigoid gestationis, and other pregnancy-related dermatoses were excluded from the analysis. In addition to the literature review, we also included a description of a patient with mucocutaneous PV treated in our department to better illustrate the clinical decision-making process. The screening and paper selection process, according to the PRISMA guideline, is presented in Figure 1.

## 3. Results

### 3.1. Findings from the Literature Review

Table 1 presents the epidemiological characteristics of the analyzed patients.

Table 2 presents the histopathological and diagnostic laboratory findings of PV in the mother and the newborn. DIF, direct immunofluorescence; ELISA, enzyme-linked immunosorbent assay.

Table 3 presents the symptoms and treatment applied in the mother and the newborn.

Due to methodological differences across all studies, including differences in ELISA kits, reporting units, and cut-off values, in all tables, no unit conversion or averaging was performed, and all laboratory results are presented as reported by the original authors.

### 3.2. Clinical Case

**Figure 2:**
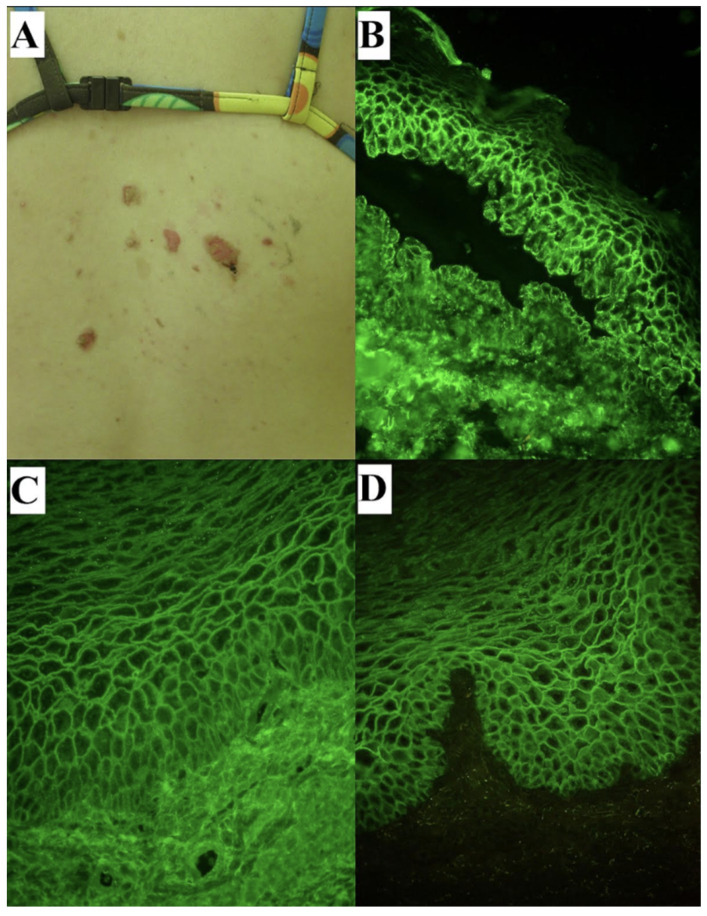
A 34-year-old female with mucocutaneous PV with initial lesions at the back, external genitals and oral mucosae illustrating the issue of PV in the context of pregnancy. Erosions with remnants of blister roofs at supraspinal skin at the time of diagnosis (**A**), made 6 months after the lesions had started. The diagnosis was established with direct immunofluorescence of perilesional skin showing pemphigus IgG4 (++) (**B**) and C3 (+) deposits in epidermis having suprabasilar separation with acantholysis, indirect immunofluorescence on monkey esophagus showing pemphigus IgG (**C**) and IgG4 (**D**) circulating antibodies both a titer above 1:80, and monovalent ELISA techniques (mv ELISA) detecting elevated levels of circulating IgG antibodies to desmoglein 3 (above 200 RU/mL, cut-off 20 RU/mL) and desmoglein 1 (28.321 RU/mL, cut-off 20 RU/mL). All photomicrographs presented were obtained using short-arc mercury lamp-operated microscopy (original objective magnification ×40). Notably, the background staining using IIFme in case of IgG4 circulating antibodies, which indicates the Th2 autoimmune response in humans, was much less intense compared to that of IgG ones. She was intravenously treated with methylprednisolone (3 times 1000 mg every other day and then 500 mg for one day; 3.5 g in total) and alternately orally with methylprednisolone 32 mg every other day. Afterwards, the dosage of oral methylprednisolone was gradually tapered and the oral dosage of 4 mg every other day controlled the disease presenting just smoldering oral lesions. At the follow-up 2 years after the diagnosis, methylprednisolone was replaced with prednisone 5 mg daily orally as she planned pregnancy. Right after the conception, IIFme showed pemphigus IgG and IgG4 antibodies both a titer above 1:80, whereas mv ELISA detected elevated levels of IgG antibodies to desmoglein 3 (above 200 RU/mL, cut-off 20 RU/mL), but levels of IgG antibodies to desmoglein 1 were within normal range. During pregnancy, her stubborn oral lesions were treated with prednisone 5 mg daily orally, which was switched to methylprednisolone 4 mg every other day after delivery. The infant was delivered by vaginal delivery, assisted with a vacuum extraction at 40 weeks of gestation with Apgar scores of 10 and complicated by postpartum hemorrhage due to an episiotomy and vaginal laceration. At birth, no skin lesions were observed; however, a few days later, transient erythematous macules appeared on the forehead. No further diagnosis was performed, and the lesions resolved spontaneously within a short period.

Clinical and laboratory findings of our patients with mc PV illustrating our recent experience in the context of PV during pregnancy are shown in Figure 2. The immunological tests were performed two years prior to pregnancy at the diagnosis and repeated right after conception.

## 4. Discussion

In this section, we mainly discuss the general issues stemmning from the presentation of published cases. Importantly, no pregnant woman with PV had a termination of pregnancy due to miscarriage or induced abortion.

In the presented cases, PV was most often diagnosed in the first trimester of pregnancy. Severe symptoms occurred in eight women. The most common manifestations were flaccid blisters and oral mucosal erosions. In all cases of AIBD, the diagnosis should be established based on direct immunofluorescence (DIF) examination of a biopsy specimen taken from clinically healthy perilesional tissue. However, in patients discussed here, histopathological examination with hematoxylin&eosin stain (H + E) was performed in all cases, while in two cases, DIF was not performed. It should be stressed here that DIF, along with the assessment of IgG4 deposits, remains the crucial laboratory diagnostic procedure [24,25,26]. The diagnosis was confirmed by performing an ELISA test, which is essential for the differentiation between PV and other AIBD. Among the newborns delivered by women suffering from PV, only two presented clinical signs of the disease. Neonatal biopsy and DIF were not performed, mainly due to the parents refusal to conduct those procedures. The diagnosis was mainly based on indirect immunofluorescence (IIF) results and the clinical presentation.

In analysing the reviewed cases, several consistent patterns emerge. Most of the reported patients developed symptoms early in pregnancy, typically between 4 and 17 weeks of gestation, suggesting a tendency for PV either to manifest de novo or worsen during the initial immunological shift toward Th2 dominance. This timing was highly comparable across studies, indicating a meaningful trend rather than incidental variation. Similarly, oral mucosal involvement was almost universally present and often accompanied with skin lesions. This is consistent with dominance of mucosal PV phenotypes in early disease and should raise clinical suspicion among dermatologists and obstetricians evaluating persistent erosive oral lesions in pregnant patients.

Our opinion is that H + E of lesional tissue has limited value in diagnosing pemphigus diseases because it cannot detect the key autoimmune phenomena that characterize their pathogenesis. Moreover, acantholysis is the pathological hallmark of pemphigus diseases, albeit not a disease-specific finding. Therefore, the general recommendation as far as tissue tests are concerned should be that diagnosing pemphigus diseases, also in the context of pregnancy, cannot be based on H + E alone [27]. All infants recovered spontaneously with only topical treatment and without oral treatment. Infants should be monitored beginning at the delivery for the clinical signs of the disease and, if lesions are stubborn, the laboratory diagnostics with DIF and multiplex ELISA should be performed. The treatment of infants showing clinical laboratory features of an active disease should be based on superpotent topical GCS applied solely on lesions, such as clobetasol propionate administered for as short a time as possible. Breastfeeding should not be recommended as transmission of maternal autoantibodies in milk is plausible. All decisions regarding infants should be made in cooperation with the neonatologist. Our review shows that systemic GCS remain the first line treatment, with prednisolone used most often because its transfer through the placenta is limited. The dosing strategies across published cases were broadly similar, beginning with moderate to high doses followed by tapering as clinical improvement permitted. In more severe or resistant cases, IVIg were added and proved to be both effective and safe. Several authors also suggested that IVIg may reduce the passage of maternal autoantibodies to the fetus, potentially explaining the generally mild neonatal symptoms. Overall, these treatment patterns are consistent across the literature and support current recommendations for managing PV during pregnancy. The pregnant women were treated with the classical method, using GCS. In two patients who had been diagnosed with PV prior to pregnancy, methylprednisolone therapy was administered before conception. Upon becoming pregnant, the medication was switched to prednisolone. When comparing the literature observations with our own case, the disease course was very similar to the patterns described. Our case therefore represents a typical example rather than an outlier and supports the conclusions drawn from the review. However, it should be emphasized that in our PV patient with smouldering oral lesions, methylprednisolone previously used chronically was replaced with prednisone just before planned conception in order to minimize fetal damage.

In general, women in reproductive age suffering from PV willing to have children should not be advised against conceiving. Modern management of PV in pregnancy should allow for delivering healthy offspring. In pregnant women diagnosed with PV, therapeutic options are limited compared to non-pregnant patients [28,29]. This limitation results primarily from the need to protect the fetus, as some drugs commonly used in the treatment of non-pregnant patients with PV may have a potentially harmful effect on fetal development [28,29]. The most common therapies used in PV are methotrexate, cyclophosphamide, and mycophenolate mofetil. All of them should be strictly avoided in pregnancy due to their high teratogenicity, as they interfere with the processes of cellular division and differentiation in the developing embryo and fetus [29,30]. Methotrexate, as a folic acid antagonist, inhibits the enzyme dihydrofolate reductase, leading to disturbances in the synthesis of proteins, RNA, and DNA. This results in severe developmental defects in the fetus [29]. Cyclophosphamide is an alkylating agent that forms cross-links within DNA. During the period of organogenesis, it also leads to the development of severe fetal malformations and may potentially cause fetal demise [29,31]. Mycophenolate mofetil (MMF) blocks inosine monophosphate dehydrogenase (IMPDH), which results in inhibition of purine synthesis in lymphocytes. During pregnancy, this leads to abnormalities in the development of mesenchymal tissues and may cause the so-called mycophenolate embryopathy syndrome [29,30].

On the other hand, there are FDA Category C drugs used in treating PV, such as topical GCS, dapsone, intravenous immunoglobulins (IVIg), rituximab, and prednisolone [28,29,32]. Topical GCS therapy in pregnant patients is considered quite safe and is usually used as a first-line treatment in cases of mild disease or as an adjunct to systemic therapy [29]. According to research findings, the use of potent topical GCS on areas of increased skin permeability, such as the eyelids, skin folds, or genital regions, is associated with a higher risk of systemic absorption [28,29]. This may lead to the occurrence of systemic adverse effects, including suppression of the hypothalamic–pituitary–adrenal axis, iatrogenic symptoms of Cushing’s syndrome, or ocular complications such as glaucoma or cataract [28,29]. Although most studies do not confirm a significant increase in the risk of congenital malformations, prolonged or extensive use of potent GCS may be associated with an increased risk of low birth weight in newborns, suggesting possible systemic absorption and its potential influence on the course of pregnancy [29,31].

The oral GCS prednisolone or, if the liver functions properly, prednisone, remain the first-line drugs in the treatment of PV during pregnancy [27,28,29,30,31]. Prednisolone is considered to be safer in pregnancy compared to other immunosuppressive medications. Due to the increased activity of the enzyme 11β-hydroxysteroid dehydrogenase type 2 (11β-HSD2) in the placenta, the active form of prednisolone is converted into inactive prednisone, and the transfer of the drug to the fetal circulation is limited to approximately 10% of the maternal concentration [28,30]. Regarding the use of methylprednisolone, a greater amount of the active drug may pass through the placenta into the fetal circulation. This is caused by the weaker activity of the enzyme 11β-HSD2 on methylprednisolone [28]. It may lead to suppression of the hypothalamic–pituitary–adrenal axis in the newborn, as well as growth disturbances or low birth weight in cases of long-term therapy [28,30]. Therefore, in patients previously diagnosed with PV who have been treated with methylprednisolone, the therapy should be switched to prednisolone at the stage of pregnancy planning [27,31].

Despite its relative safety, dapsone is not commonly used in PV [23,27]. The use of dapsone during pregnancy is primarily associated with the risk of red blood cell hemolysis in both the mother and the fetus, as the drug crosses the placenta [23,27]. In addition, in women with glucose-6-phosphate dehydrogenase (G6PD) deficiency, it may lead to severe hemolytic anemia [23,27]. IVIg are usually used as a second-line treatment in cases where there is insufficient improvement after corticosteroid therapy or when contraindications to their use are present [23,27]. In pregnant women, high doses of 2 g/kg body weight administered over five days are used, with treatment cycles repeated monthly during pregnancy and in the first 2–3 months after delivery [30]. Most importantly, it has been observed that IVIg prevents the transplacental transfer of autoantibodies from the maternal to the fetal circulation [30,31], which may potentially reduce the incidence of neonatal PV [32,33].

Recently, rituximab has become a standard therapy in non-pregnant women affected by PV [34,35]. Rituximab is a chimeric monoclonal antibody directed against the CD20 antigen on B lymphocytes [34]. The drug shows a very long duration of action and may suppress B cells for up to 12 months after administration [36]. Rituximab crosses the placental barrier to varying degrees during pregnancy, which is related to maturation of chorionic villi and placental Fc receptors; in the first trimester, owing to absent/immature Fc receptor–mediated transport, fetal exposure is minimal, whereas transplacental transfer increases with gestational age and peaks in late third trimester [37,38,39]. No consistent teratogenic signal has been demonstrated in humans, and animal studies have not shown structural malformations attributable to rituximab [37]. However, according to the product labeling, rituximab administration should be discontinued 12 months before a planned conception [36]; nevertheless, these recommendations are not always fulfilled.

There are reports describing the use of immunotherapy during pregnancy, although data on the treatment of PV are scarce. The largest report published in 2022 included 19 women exposed to rituximab either within 12 months before conception or during pregnancy; 14 had PV. In 89% of the cases, the pregnancy resulted in a healthy live birth, and preterm delivery occurred in 17%; no congenital anomalies were observed [40]. Another report and the case literature describe neonatal immune effects after in utero exposure, including transient B-cell depletion, hypogammaglobulinemia and, rarely, neonatal sepsis [40,41]. In women of child-bearing potential who are not planning pregnancy, effective contraception is recommended during therapy and for 12 months after the last dose [36,42]; some authors prefer non-hormonal methods, given potential hormonal influences on PV activity [43].

As for non-hormonal methods, regardless of the PV treatment used, barrier contraception seems to be a useful option. Nevertheless, concerns may arise that the diaphragm may induce the Köbner phenomenon, also known as the isomorphic response triggering the vaginal lesions in women with clinically active PV [44]. In this respect, non-penetrative sex can be a recommended option. The counselling of sexual partners may be important in understanding the potential impact of sexual activity in the context of PV pathogenesis. Non-penetrative sexual practices may reduce cervicovaginal microtrauma, which can exacerbate disease course and complicate management.

This review has several limitations. The available evidence is restricted to single case reports and small case series, which limits the strength of the conclusions. Diagnostic methods and antibody assays were not fully uniform across the publications, and in several reports treatment descriptions lacked detail. Despite these limitations, the available evidence indicates that with appropriate interdisciplinary management, most women with PV can achieve favourable pregnancy outcomes.

## 5. Conclusions

Management of PV in pregnancy requires the cooperation of dermatologist, gynecologistobstetrician and neonatologist. All of them should be aware of peculiarities of PV. Young women already suffering from PV before a planned pregnancy require careful specialist and educational attention.

If PV occurs during pregnancy, treatment should be chosen with a priority focus on the safety of the fetus. Moreover, due to the increasing use of rituximab in PV nowadays, pregnancy should be postponed for at least 12 months after therapy completion in order to minimize the potential risk of immunosuppression in the newborn. Perhaps then, pregnancy-related pemphigus diseases, due to their peculiarities, should be added to the list of distinct varieties within the desmosomal type of AIBD. This speculation is not currently evidence-based. As such, it requires more comparative clinical–epidemiological research, which, hopefully, will provide convincing empirical data for its validation.

## Figures and Tables

**Table 1:** Epidemiology of cases.

Authors	Diagnosis of PV Before Conception	Number of Cases	Women Age	Gravida and Para	Gestational Week of First Symptoms	Symptoms in Mother	Symptoms in Neonatal
Sanmitra Aiholliv [15]	No	1	29	Not reported	4-month	Yes	Yes
Ayush Anand [16]	No	1	28	G2P2	12 weeks	Yes	Yes
Yasuhiro Kano [17]	No	1	39	G2P2	14 weeks	Yes	No
Fatemeh Mohaghegh [18]	No	1	41	G5P4	4 weeks	Yes	No
Ecem Bostan [19]	No	1	32	G4G4	1-month	Yes	Not reported
Eric R Bray [20]	No	1	34	G4P3	17 weeks	Yes	Not reported
Rıfkiye Küçükoğlu [21]	No and Yes	2	23 and 28	G1P1 and G1P1	8 weeks and 12 weeks	Yes and Yes	No and no
Harina Vin [22]	Yes	1	29	G1P1	7 weeks	Yes	No
Javier Rangel [23]	No	1	24	G1P1	15 weeks	Yes	No

**Table 2:** Histopathology and diagnostic of PV in pregnancy.

Authors	Histopathology	DIF	ELISA	Histopathology in Neonate	DIF in Neonate	ELISA in Neonate
Sanmitra Aiholli [15]	Suprabasal split overlying a row of basal keratinocytes, attached to the basement membrane, resembling tombstones.	Epidermal intercellular staining with IgG and C3	Positive for Dsg1 and Dsg3 antibodies at a titre of 1:10	Not performed	Not performed	Positive for Dsg1 and Dsg3 antibodies at a titre of 1:10
Ayush Anand [16]	Suprabasal cleft, acantholysis, tombstone basal layer.	Intercellular IgG in a fish-net pattern	Not performed	Suprabasal cleft, acantholysis, tombstone basal layer	Intercellular IgG in a fish-net pattern	Not performed
Yasuhiro Kano [17]	Intraepidermal vesicular dermatitis with suprabasal cleft and acantholytic cells.	Not performed	Elevated Dsg1 = 109 U/mL, Dsg3 = 155 U/mL	Not performed	Not performed	Not performed
Fatemeh Mohaghegh [18]	Suprabasal bulla with “tombstone” basal layer; acantholysis; dermal lymphocytes ± eosinophils	Not performed	Anti-Dsg1 1:32, anti-Dsg3 1:320	Not performed	Not performed	Not performed
Ecem Bostan [19]	Suprabasal acantholysis and eosinophil-rich superficial perivascular, interstitial inflammation in dermis	Intraepidermal IgG-positive	Not performed	Not reported	Not reported	Not reported
Eric R Bray [20]	Prominent suprabasal acantholysis, tombstoning, marked eosinophilia (epidermis/superficial dermis)	Intercellular C3 and IgG in epidermis	Anti-Desmoglein 1- and 3-positive; Varicella IgG-positive	Not reported	Not reported	Not reported
Rıfkiye Küçükoğlu [21]	Emphasis on eosinophil-predominant infiltrate.	Intercellular IgG and C3 in the epidermis	Anti-Dsg1- and anti-Dsg3-positive	Not performed	Not performed	Not performed
Harina Vin [22]	Subepidermal detachment with eosinophils/neutrophils	Linear C3 along dermal–epidermal junction.	BP180 = 76 (ref < 9), BP230-negative	Not performed	Not performed	Not performed
Javier Rangel [23]	Suprabasal and intraepidermal acantholysis	Intercellular IgG + C3+	Dsg1 = 65 U, Dsg3 = 285 U	Not performed	Not performed	Not performed

**Table 3:** Symptoms and treatment applied.

Authors	Symptoms of PV in Mother	Treatment Applied	Symptoms of PV in Neonate	Treatment Applied in Neonate
Sanmitra Aiholli [15]	Flaccid blisters over scalp, oral cavity and trunk, which ruptured to form erosions and crusted plaques	Prednisolone 20 mg/day, reduced to 5 mg/day	Blisters and erosions over trunk and extremities.	Not reported
Ayush Anand [16]	Flaccid bullae and erosions over scalp, face, abdomen, upper/lower limbs, oral cavity	Prednisolone 40 mg/day then 10 mg/day over 6 months + local wound care; later switched to azathioprine 50 mg p.o daily	Blistering and vesicles on neck, earlobes, foot	Topical mupirocin + local wound care
Yasuhiro Kano [17]	Periumbilical rash with erythema and blisters, later spreading around the navel and over most of the body	Prednisolone 50 mg/day orally. Poor response → IV methylprednisolone 1 g/day × 3 days (two pulses) → IV immunoglobulin (IVIg)	Healthy infant	Healthy infant
Fatemeh Mohaghegh [18]	Flaccid blisters and vesicles on abdomen, chest, arms, back, oral (buccal) and vulvar mucosa involved	Prednisolone 65 mg/day started at diagnosis → partial response in 4 weeks → tapered gradually to 20 mg/day by 5 months; continued low-dose prednisolone postpartum	Healthy infant	Healthy infant
Ecem Bostan [19]	Mucocutaneous blister with crusting, erosion, and ulceration, vesicles and bullae all over the trunk	Prednisolone 30 mg/day and 120 g IVIG treatment	Not reported	Not reported
Eric R Bray [20]	Pruritic diffuse vesicles/bullae and erosions on chest, abdomen, back, buttocks, thighs, arms, oral mucosal erosions (buccal/labial/tongue)	Initial topical corticosteroids + empiric acyclovir → then IV methylprednisolone 20 mg × 3 doses → oral prednisone 60 mg mg/day	Not reported	Not reported
Rıfkiye Küçükoğlu [21]	1. Severe pruritus, oral erosions, and annular vesiculobullous plaques, 2. Extensive polycyclic plaques encircling crusted lesions; severe pruritus; mucosa spared	1. Systemic corticosteroids, plasmapheresis, IVIG 0.4 g/kg/day for 5 consecutive days, achieving remission. 2. Before pregnancy methylprednisolone, during pregnancy systemic corticosteroids were not sufficient; advanced modalities	1. Healthy infant and 2. Healthy infant	Healthy infant
Harina Vin [22]	Oral erosions in 7th week. Two weeks before delivery: intense pruritus (umbilicus, inner thighs, hands). After delivery: tense vesicles/bullae on erythematous base on abdomen, medial thighs, and several on upper/lower extremities	Prednisone + mycophenolic acid → switched to azathioprine. At ~7 weeks’ GA, azathioprine stopped; no systemic immunosuppression during pregnancy	Healthy infant	Healthy infant
Javier Rangel [23]	Crusted erosions, areas of denuded epidermis, and scattered flaccid and clear fluid-filled bullae confined to the trunk and extremities.	Prednisone 30 mg/day + topical steroids. Postpartum: persistent activity → rituximab as steroid-sparing; remission at last follow-up	Healthy infant	Healthy infant

## Data Availability

The authors confirm that the data supporting the findings of this study are available within the article.

## References

[1] Bystryn J.-C., Rudolph J.L. (2005). Pemphigus. Lancet.

[2] Freedberg I.M., Fitzpatrick T.B. (1999). Fitzpatrick’s Dermatology in General Medicine.

[3] Dmochowski M., Jałowska M., Bowszyc-Dmochowska M. (2022). Issues Occupying Our Minds: Nomenclature of Autoimmune Blistering Diseases Requires Updating, Pemphigus Vulgaris Propensity to Affect Areas Adjacent to Natural Body Orifices Unifies Seemingly Diverse Clinical Features of This Disease. Front. Immunol..

[4] Ahmed A.R., Yunis E.J., Khatri K., Wagner R., Notani G., Awdeh Z., Alper C.A. (1990). Major Histocompatibility Complex Haplotype Studies in Ashkenazi Jewish Patients with Pemphigus Vulgaris. Proc. Natl. Acad. Sci. USA.

[5] Ahmed A.R., Wagner R., Khatri K., Notani G., Awdeh Z., Alper C.A., Yunis E.J. (1991). Major Histocompatibility Complex Haplotypes and Class II Genes in Non-Jewish Patients with Pemphigus Vulgaris. Proc. Natl. Acad. Sci. USA.

[6] Loiseau P., Lecleach L., Prost C., Lepage V., Busson M., Bastuji-Garin S., Roujeau J.-C., Charron D. (2000). HLA Class II Polymorphism Contributes to Specify Desmoglein Derived Peptides in Pemphigus Vulgaris and Pemphigus Foliaceus. J. Autoimmun..

[7] Fagundes P.P.S., Santi C.G., Maruta C.W., Miyamoto D., Aoki V. (2021). Autoimmune Bullous Diseases in Pregnancy: Clinical and Epidemiological Characteristics and Therapeutic Approach. An. Bras. Dermatol..

[8] Saito S., Nakashima A., Shima T., Ito M. (2010). REVIEW ARTICLE: Th1/Th2/Th17 and Regulatory T-Cell Paradigm in Pregnancy. Am. J. Reprod. Immunol..

[9] Fan H., Zhao G., Liu L., Liu F., Gong W., Liu X., Yang L., Wang J., Hou Y. (2012). Pre-Treatment with IL-1β Enhances the Efficacy of MSC Transplantation in DSS-Induced Colitis. Cell Mol. Immunol..

[10] Bialynicki-Birula R., Dmochowski M., Maj J., Gornowicz-Porowska J. (2011). Pregnancy-Triggered Maternal Pemphigus Vulgaris with Persistent Gingival Lesions. Acta Dermatovenerol. Croat..

[11] Kridin K., Schmidt E. (2021). Epidemiology of Pemphigus. JID Innov..

[12] Hup J.M., Bruinsma R.A., Boersma E.R., de Jong M.C.J.M. (1986). Neonatal Pemphigus Vulgaris: Trsmtel Transmission of Antibodies. Pediatr. Dermatol..

[13] Bjarnason B., Flosadóttir E. (1999). Childhood, Neonatal, and Stillborn Pemphigus Vulgaris. Int. J. Dermatol..

[14] Hilario-Vargas J., Dasher D.A., Li N., Aoki V., Hans-Filho G., dos Santos V., Qaqish B.F., Rivitti E.A., Diaz L.A. (2006). Prevalence of Anti-Desmoglein-3 Antibodies in Endemic Regions of Fogo Selvagem in Brazil. J. Investig. Dermatol..

[15] Aiholli S., Inamadar A., Janagond A.B. (2024). Neonatal Pemphigus Vulgaris. BMJ Case Rep..

[16] Anand A., Awake P., Bhosale A., Bindu R., Kher A., Bhanap P. (2023). Pemphigus Vulgaris in a Neonate Born to a Mother with Pemphigus Vulgaris: A Case Report. Clin. Case Rep..

[17] Kano Y., Kato M. (2023). Periumbilical Blisters: Pemphigus Vulgaris During Pregnancy. Am. J. Obstet. Gynecol..

[18] Mohaghegh F., Shahmoradi Z., Alizadeh M. (2022). Pregnancy-Triggered Pemphigus Vulgaris with Favorable Fetal Outcomes: A Case Report. Clin. Case Rep..

[19] Bostan E., Gülseren D., Ersoy Evans S., Elçin G., Karaduman A., Atakan N. (2020). Efficacious Treatment of Pemphigus Vulgaris by Intravenous Immunoglobulin During Pregnancy and Postpartum Period. Dermatol. Ther..

[20] Bray E.R., Bray F.N., Herskovitz I., Cho-Vega J.H. (2020). New Onset Bullae in a Pregnant Woman. Int. J. Dermatol..

[21] Küçükoğlu R., Sun G.P., Kılıç S. (2018). Polycyclic Annular Presentation of Pemphigus Vulgaris with an Eosinophil Predominance in Two Pregnant Patients. Dermatol. Online J..

[22] Vin H., Seyfer S.J., McClain C.M., Hsu S. (2016). Concomitant Pemphigus Vulgaris and Pemphigoid Gestationis: A Case Report and Review of the Literature. Dermatol. Online J..

[23] Rangel J. (2016). Pregnancy-Associated “Cutaneous Type” Pemphigus Vulgaris. Perm J..

[24] Garbanzos C.C.T., Todd A., Hardway H.D., Lehman J.S. (2025). Clinical, Serologic, and Histopathologic Features of Patients With Pemphigus With Either Positive or Negative IgG4 Intercellular Deposition by Direct Immunofluorescence (DIF): A Retrospective Case–Control Study of 55 DIF Biopsy Specimens. J. Cutan Pathol..

[25] Jałowska M., Falkowski B., Bowszyc-Dmochowska M., Raptis-Bolwach M., Gornowicz-Porowska J., Seraszek-Jaros A., Dmochowski M. (2025). Effectiveness of Direct Immunofluorescence for Diagnosing Autoimmune Bullous Diseases: Data from the Central European Referral Department. Adv. Dermatol. Allergol..

[26] Kim R.H., Brinster N.K. (2020). Practical Direct Immunofluorescence. Am. J. Dermatopathol..

[27] Jałowska M., Bowszyc-Dmochowska M., Spałek M., Falkowski B., Dmochowski M. (2023). Pemphigus Foliaceus in an Elderly Woman: Advantages and Limitations of Histopathology in Pemphigus. Dermatol. Rev..

[28] Lehman J.S., Mueller K.K., Schraith D.F. (2008). Do Safe and Effective Treatment Options Exist for Patients With Active Pemphigus Vulgaris Who Plan Conception and Pregnancy?. Arch. Dermatol..

[29] Genovese G., Derlino F., Berti E., Marzano A.V. (2020). Treatment of Autoimmune Bullous Diseases During Pregnancy and Lactation: A Review Focusing on Pemphigus and Pemphigoid Gestationis. Front. Pharmacol..

[30] Tavakolpour S. (2017). Current and Future Treatment Options for Pemphigus: Is It Time to Move Towards More Effective Treatments?. Int. Immunopharmacol..

[31] Kridin K., Hammers C., Ludwig R., Bitan D., Cohen A. (2021). Survival of Adjuvant Drugs for Treatment of Pemphigus: A Population-Based Cohort Study. Acta Derm. Venereol..

[32] Ahmed A.R., Gürcan H.M. (2011). Use of Intravenous Immunoglobulin Therapy During Pregnancy in Patients with Pemphigus Vulgaris. J. Eur. Acad. Dermatol. Venereol..

[33] Kianfar N., Dasdar S., Mahmoudi H., Daneshpazhooh M. (2022). Burden of Pemphigus Vulgaris with a Particular Focus on Women: A Review. Int. J. Womens Dermatol..

[34] Joly P., Horvath B., Patsatsi A., Uzun S., Bech R., Beissert S., Bergman R., Bernard P., Borradori L., Caproni M. (2020). Updated S2K Guidelines on the Management of Pemphigus Vulgaris and Foliaceus Initiated by the European Academy of Dermatology and Venereology (EADV). J. Eur. Acad. Dermatol. Venereol..

[35] Werth V.P., Joly P., Mimouni D., Maverakis E., Caux F., Lehane P., Gearhart L., Kapre A., Pordeli P., Chen D.M. (2021). Rituximab versus Mycophenolate Mofetil in Patients with Pemphigus Vulgaris. N. Engl. J. Med..

[36] Miše J., Jukić I.L., Marinović B. (2022). Rituximab—Progress but Still Not a Final Resolution for Pemphigus Patients: Clinical Report From a Single Center Study. Front. Immunol..

[37] Perrotta K., Kiernan E., Bandoli G., Manaster R., Chambers C. (2021). Pregnancy Outcomes Following Maternal Treatment with Rituximab Prior to or during Pregnancy: A Case Series. Rheumatol. Adv. Pr..

[38] Gklinos P., Dobson R. (2023). Monoclonal Antibodies in Pregnancy and Breastfeeding in Patients with Multiple Sclerosis: A Review and an Updated Clinical Guide. Pharmaceuticals.

[39] Beltagy A., Aghamajidi A., Trespidi L., Ossola W., Meroni P.L. (2021). Biologics During Pregnancy and Breastfeeding Among Women With Rheumatic Diseases: Safety Clinical Evidence on the Road. Front. Pharmacol..

[40] Dehghanimahmoudabadi A., Kianfar N., Akhdar M., Dasdar S., Balighi K., Mahmoudi H., Daneshpazhooh M. (2022). Pregnancy Outcomes in Women with Pemphigus Exposed to Rituximab Before or During Pregnancy. Int. J. Womens Dermatol..

[41] Klink D.T., van Elburg R.M., Schreurs M.W.J., van Well G.T.J. (2008). Rituximab Administration in Third Trimester of Pregnancy Suppresses Neonatal B-Cell Development. Clin. Dev. Immunol..

[42] Flint J., Panchal S., Hurrell A., van de Venne M., Gayed M., Schreiber K., Arthanari S., Cunningham J., Flanders L., Moore L. (2016). BSR and BHPR Guideline on Prescribing Drugs in Pregnancy and Breastfeeding—Part I: Standard and Biologic Disease Modifying Anti-Rheumatic Drugs and Corticosteroids: Table 1. Rheumatology.

[43] De D., Shah S., Mahajan R., Handa S. (2024). Pemphigus and Pregnancy. Indian Dermatol. Online J..

[44] Daneshpazhooh M., Fatehnejad M., Rahbar Z., Balighi K., Ghandi N., Ghiasi M., Abedini R., Lajevardi V., Chams-Davatchi C. (2016). Trauma-induced Pemphigus: A Case Series of 36 Patients. JDDG J. Dtsch. Dermatol. Ges..

